# Percepción de los profesionales de enfermería sobre la aplicabilidad del proceso de continuidad de cuidados*

**DOI:** 10.15649/cuidarte.2210

**Published:** 2023-05-27

**Authors:** Karen Liseth Rojas-Manzano, Nicolay Toro-Delgado, Deysi Johana Eraso-Riascos, Edna Johana Mondragón-Sánchez

**Affiliations:** 1 Universidad del Quindío, Quindío, Colombia. Email: karenroman1309@gmail.com Universidad del Quindío Universidad del Quindío Quindío Colombia karenroman1309@gmail.com; 2 Universidad del Quindío, Quindío, Colombia. Email: ntorod@uqvirtual.edu.co Universidad del Quindío Universidad del Quindío Quindío Colombia ntorod@uqvirtual.edu.co; 3 Universidad del Quindío, Quindío, Colombia. Email: deysieraso@gmail.com Universidad del Quindío Universidad del Quindío Quindío Colombia deysieraso@gmail.com; 4 Universidad del Quindío, Quindío, Colombia. Miembro del Grupo de Atención Primaria en Salud GIAPS. Email: ejmondragon@uniquindio.edu.co Universidad del Quindío Universidad del Quindío Quindío Colombia ejmondragon@uniquindio.edu.co

**Keywords:** Continuidad de la Atención al Paciente, Educación en Enfermería, Enfermeras y Enfermeros, Pandemia, Continuity of Patient Care, Education in Nursing, Nurses and Nurses, Pandemic, Continuidade dos Cuidados com o Doente, Educação em Enfermagem, Enfermeiros e Enfermeiros, Pandemia

## Abstract

**Introducción:**

La Continuidad de Cuidados se comprende a partir de la adaptación del sujeto de cuidado y su red de apoyo a la nueva situación de salud-enfermedad. Objetivo: Comprender la percepción de los profesionales de enfermería sobre la aplicabilidad del proceso de continuidad de cuidados.

**Materiales y Método:**

Estudio con abordaje cualitativo, fenomenológico, los sujetos de estudio se conformaron por profesionales de enfermería coordinadores de área, realizando entrevistas semiestructuradas, para el análisis de los datos se utiliza el software AtlasT.

**Resultados:**

Se encontró que la continuidad de cuidados se aplica parcialmente por los participantes, esto debido a la carencia de claridad del concepto, la desarticulación entre atención primaria en salud, atención especializada y los limitantes administrativos; así mismo, esta es practicada de forma desorganizada y de forma empírica.

**Discusión:**

Es evidente la necesidad de la aplicación de estrategias en pro de la comunicación entre los diferentes niveles de atención, para fortalecer el trabajo en equipo y favorecer el autocuidado de los pacientes, dado que a consideración de los participantes la información no es compartida entre los diferentes niveles de atención y esto dificulta la continuidad de cuidados individual de los pacientes.

**Conclusiones:**

La aplicación de la Continuidad de Cuidados es fragmentada y desordenada, además de esto, se reconoce que, con base en la evidencia de otros países, si dicho proceso se aplicara en Colombia mejoraría la calidad del servicio y así mismo la calidad de vida de los pacientes.

## Abstract (en)

**Introduction:**

The continuity of care is understood as the adaptation of caregivers and their support network to the new health-disease situation.

**Objective:**

To understand nurses’ perception of the applicability of the continuity of care process.

**Materials and Methods:**

Study with a qualitative and phenomenological approach. The subjects of the study were nurse coordinators. Semi-structured interviews were conducted. Atlas.ti was used for data analysis. Results: The participants partially applied continuity of care due to a lack of clarity of the concept, disconnection between primary health care and specialized care, and administrative limitations. The continuity of care is practiced in a disorganized and empirical manner.

**Discussion:**

There is a clear need for implementing communication strategies between the different levels of care to strengthen teamwork and promote patient self-care. According to the participants, the information is not shared among the different levels of care, hindering the continuity of patient care.

**Conclusions:**

The application of the continuity of care process is fragmented and disorganized. Based on evidence from other countries, the participants recognized that if continuity of care were applied in Colombia, itwouldimprovethequalityofservicesandthequalityoflifeofpatients.

## Resumo

**Introdução:**

A Continuidade do Cuidado é compreendida a partir da adaptação do sujeito do cuidado e sua rede de apoio à nova situação de saúde-doença. Objetivo: Compreender a percepção dos profissionais de enfermagem sobre a aplicabilidade do processo de continuidade do cuidado.

**Materiais e Método:**

Estudo com abordagem qualitativa, fenomenológica, os sujeitos do estudo foram constituídos por profissionais de enfermagem coordenadores da área, realizando entrevistas semiestruturadas, para análise dos dados foi utilizado o software AtlasT.

**Resultados:**

Constatou- se que a continuidade do cuidado é parcialmente aplicada pelos participantes, devido à falta de clareza do conceito, à desarticulação entre atenção básica, atenção especializada e limitações administrativas; da mesma forma, isso é praticado de forma desorganizada e empírica.

**Discussão:**

Fica evidente a necessidade de aplicação de estratégias em prol da comunicação entre os diferentes níveis de atenção, para fortalecer o trabalho em equipe e favorecer o autocuidado dos pacientes, haja vista que, na opinião dos participantes, as informações não são compartilhadas entre os diferentes níveis de atenção e isso dificulta a continuidade do cuidado individual ao paciente.

**Conclusões:**

A aplicação da Continuidade do Cuidado é fragmentada e desordenada, além disso, reconhece- se que, com base em evidências de outros países, se esse processo fosse aplicado na Colômbia, melhoraria a qualidade do serviço e também a qualidade do atendimento. vida dos pacientes.

## Introducción

La Joint Comission on Accreditation of Health Care Organizations (JCAHCO) define la Continuidad de Cuidados (CC) como «el grado en que la asistencia que necesita el paciente está coordinado eficazmente entre diferentes profesionales y organizaciones, con relación al tiempo», teniendo como objetivo definir, dar forma, ordenar los procesos y actividades para potenciar al máximo la coordinación dentro de la continuidad asistencial^1^^,^^2^. Sin embargo, no solo se constituye en un soporte asistencial y de transferencia de información en salud como método de reducción de obstáculos y mejora de los cuidados integrales que se brindan. Así, el fenómeno de la CC se comprende a partir de la adaptación del sujeto de cuidado y su red de apoyo a la nueva situación de salud-enfermedad, la disminución de la incertidumbre ante esta, la contemplación de todos los actores sanitarios para la mejora y autonomía del sujeto, la inclusión de los entornos institucionales y comunitarios como pilares de la atención en salud^1^^,^^3^.

La CC es, por lo tanto, un proceso compuesto de elementos como personas (pacientes y talento humano en salud), entornos (institucionales y extrainstitucionales) e información (verbal y escrita) para brindar la atención a persona/familia/cuidadores a través de unas herramientas (historia clínica, comisiones de cuidado de área, guías de CC, redes de investigación, entre otras), así como los instrumentos de valoración del Grado de Continuidad (CTM-3M), los cuales evalúan la implementación en un lugar específico; y los procesos evaluativos como métodos de mejora como el ciclo de la calidad de Deming o ciclo Planificar-Ejecutar-Chequear-Actuar (PDCA)^1^^,^^4^^,^^5^.

A nivel mundial el interés en la CC aumenta paulatinamente con acercamientos a los pacientes a través de algunas iniciativas como la planificación exhaustiva del plan de alta, la comunicación de la situación de salud entre instituciones que atienden un mismo paciente y la gestión de los servicios de salud desde la perspectiva de la cultura organizacional como respuesta a la calidad de los servicios^6^^,^^7^^,^^8^.

Los países que citan la CC, implementan y evalúan su aporte se remiten mayoritariamente a México^2^, Chile^6^, España^7^ y Portugal^8^. En Colombia, seen contraronpocas referencias acercadela CC, lo encontrado se constituye en los hallazgos acerca de la importancia de la comunicación y coordinación de los profesionales de enfermería en servicios de alta complejidad^8^. Se tiene, por lo tanto, el panorama del abordaje e investigación deficitaria, lo que argumenta, incluso más, la necesidad de la investigación en salud sobre la CC.

Las instituciones de salud resaltan la importancia de la CC como base fundamental de los servicios prestados^10^. Por lo cual, y ante su importancia como elemento clave en la calidad, la CC se transforma en un reto por diversos motivos, estas razones justifican la importancia de profundizar en el estudio de la misma, la comprensión de las situaciones internas de los sistemas de salud que posibiliten y/o dificulten la práctica de ella y cómo estas se pueden avocar hacia la calidad en los cuidados que propende la CC. Estableciéndose como táctica del mejoramiento del acceso a los servicios de salud de manera oportuna, eficaz y con calidad para la preservación, el mejoramiento y la promoción de la salud^11^, atendiendo al objetivo de la política en salud en Colombia, propósito concomitante con la CC.

Es importante resaltar que el valor fundamental para el funcionamiento de la CC es la comunicación, componente que permite el flujo adecuado de datos y apreciaciones interdisciplinarias posibilitando el abordaje integral del sujeto de cuidado^1^^,^^3^^).^

Los profesionales de enfermería, líderes en la gestión del cuidado^12^, demuestran a través de las experiencias compartidas las mejoras en la coordinación y el seguimiento de los pacientes a través de la reestructuración del sistema de salud fundamentados en la CC, dando cumplimiento a uno de los principios de la práctica profesional: Continuidad y calidad como un deber de la disciplina^13^^,^^14^ ya que la planificación de los cuidados con relación a las necesidades y problemáticas de la población como en la actualidad la pandemia, influye en la mejora de la calidad y contribuye a la resolución de los problemas^2^.

La consumación de lo anterior implica un impacto en los defectos de la calidad y la oportunidad por demoras, trámites y remisiones innecesarias, que perjudican los resultados en salud de las personas, además de la credibilidad y confianza de los pacientes frente a las instituciones y la idoneidad del talento humano^15^.

Asimismo, reconociendo al profesional de enfermería como el profesional idóneo por su cualificación y educación universitaria tendiente a la gestión del cuidado y liderazgo^12^, la coordinación, implementación y evaluación de los sistemas de salud, se propone a estos como los indicados para liderar los procesos de CC en todos los niveles.

Dada la evidencia científica que avala la importancia de la CC como proceso sanitario de calidad en todos los servicios brindados, es necesario por consiguiente, comprender desde la perspectiva de los profesionales del cuidado en el contexto colombiano la aplicabilidad del proceso de CC, es por esto que para la mejor comprensión de los diferentes fenómenos, se contempló los conceptos de satisfacción y percepción para dar respuesta al objetivo de investigación: 1. Percepción^16^ es biocultural porque, por un lado, depende de los estímulos físicos y sensaciones involucrados y, por otro lado, de la selección y organización de dichos estímulos y sensaciones; las experiencias sensoriales se interpretan y adquieren significado moldeadas por pautas culturales e ideológicas específicas aprendidas desde la infancia^16^. 2. Satisfacción de un consumidor es el resultado de comparar su percepción de los beneficios que obtiene, con las expectativas que tenía de recibirlos. Si este concepto se expresara de forma matemática se tendría lo siguiente: Satisfacción = Percepciones - Expectativas^17^. Objetivo: Comprender la percepción de los profesionales de enfermería sobre la aplicabilidad del proceso de continuidad de cuidados.

## Materiales y Métodos

Estudio con abordaje cualitativo, sustentado desde el enfoque fenomenológico de Martin Heidegger, que permite observar sin prejuicios, desvelar y comprender el fenómeno del profesional de enfermería como un ente indivisible, singular y único en el mundo que vive siente y percibe de manera singular y propia^18^.

En Colombia existe pocas referencias acerca de a CC, lo encontrado se constituye en los hallazgos acerca de la importancia de la comunicación y coordinación de los profesionales de enfermería, por lo cual teniendo en cuenta que independientemente del nivel en el que trabaje el profesional de enfermería, hace parte de la red del proceso de CC9. Así teniendo presente esta literatura los participantes fueron profesionales de enfermería que cumplieron los siguientes criterios de inclusión: ser profesionales de enfermería coordinadores de área y aquellos que aceptaran participar en el estudio con previa explicación del objetivo de la investigación. Fueron en total seis profesionales de enfermería: un coordinador de área comunitaria, un coordinador administrativo y cuatro coordinadores de área clínica.

En la recolección de la información, se realizaron entrevistas semiestructuradas con un guion de preguntas, las cuales estaban orientadas a hacer aflorar en la conversación los fenómenos a evaluar: la percepción de la aplicabilidad del proceso de CC en la práctica enfermera individual, por un lado, la percepción de la aplicación de la misma en el gremio enfermero, por el otro y la percepción de los profesionales de enfermería sobre la satisfacción de las necesidades del sujeto de cuidado con la práctica enfermera, siendo estos diálogos entre febrero y mayo del 2018 en el lugar de trabajo, asegurando un espacio de tranquilidad y aislamiento de terceros. Las entrevistas fueron grabadas en audio con un promedio de duración de 1 a 2 horas cada una. El muestreo fue por medio del método bola de nieve hasta saturación de los datos^19^. Se realizó, de igual manera, un diario de campo tras terminar cada entrevista, con el objetivo de consignar información reflexiva del encuentro que proporcionó ayuda en el estudio posterior de los datos.

El análisis de la información se realizó por medio del Análisis de Contenido de Bardin^20^, utilizando para este análisis el software AtlasT 9 con la Licencia No. L-24E-AD0, el método de Bardin ayudo en el descubrimiento de las percepciones partiendo de los núcleos de los sentidos que componen una comunicación; la primera etapa de pre-análisis se realizó una lectura de los discursos, cada una de las entrevistas se escucharon de manera directa de las grabadoras para identificar la calidad de la misma y posteriormente se volvían a escuchar para transcribirlas en el procesador de Word. Es importante mencionar que cada entrevista se iba transcribiendo conforme se finalizaban las mismas de tal manera que pudieran dar claridad de información a los investigadores y, en caso necesario, volver con el entrevistado si alguna información requería un abordaje nuevo por comprensión o profundización.

La segunda etapa de exploración del material, consistente en la construcción de las operaciones de la codificación. De esta manera se fueron identificando las unidades de significado para configurar las categorías extraídas hasta la saturación de la información^20^.

En la última etapa es el tratamiento de los resultados e interpretación, se realizó el análisis de las categorías construidas para poder comprender la percepción de los profesionales de enfermería sobre la aplicabilidad del proceso de CC.

Los principios éticos de esta investigación se fundamentaron en la declaración de Singapur, la ley 1098 de 2006 y la resolución 008430 de la República de Colombia, con aprobación del Comité de Ética de la Facultad de Ciencias de la Salud de la Universidad del Quindío con el Acta N° 08 con la fecha de 13 de diciembre de 2017. Los investigadores son estudiantes del Grupo de Investigación de Atención Primaria de Salud (GIAPS), reconocido por Colciencias; adjunto a esto, los participantes firmaron el consentimiento informado tras lectura y explicación del mismo con el despeje de dudas pertinente, estos se custodian como documentos confidenciales. Se considera importante mencionar que el manuscrito fue realizado teniendo en cuenta la lista de chequeo COREQ para investigaciones cualitativas.

## Resultados

Partiendo de los discursos de los participantes y así dando respuesta a el objetivo de comprender la percepción de los profesionales de enfermería sobre la aplicabilidad del proceso de CC, se organizó los resultados en tres categorías centrales: Realidades de la práctica CC: Barreras y Dificultades; Percepción de los enfermeros con relación a la satisfacción de necesidades del paciente con la CC; y Relación de la percepción individual y la percepción colectiva sobre la CC.

Realidades de la Práctica Continuidad de Cuidados:

*Barreras y Dificultades:* La CC se practica en el contexto parcialmente, esta inexistencia integral radica en tres puntos: la carencia de claridad del proceso de CC, la desarticulación entre la APS y Atención Especializada (AE) y los limitantes administrativos.

La contemplación del paciente y cuidadores en el proceso de salud-enfermedad, la información dada de carácter verbal, el uso de lugares extrainstitucionales donde se brinda atención en salud y las herramientas utilizadas como la historia clínica son los que se manifiestan en la práctica enfermera evaluada.

Sin embargo, la CC como conocimiento epistemológico previo no es reconocida; los usos de los ítems nombrados anteriormente se hacen por motivos de seguimiento del paciente si presenta clasificación de alto riesgo, por ejemplo, las gestantes o pacientes con cronicidad o por motivos de educación tras el alta:

*“[…] una profesora nos decía que debíamos ver más allá al paciente después de una estancia hospitalaria, de pronto hacerle un seguimiento como pudiéramos para poder hacer mucho más satisfactoria esa recuperación, pero fue más como a nivel personal no a nivel de materia”: E1.*

La desarticulación entre APS y AE, por la deficiente comunicación entre profesionales interniveles o en el mismo nivel si el paciente cambia de profesional produce que la información no fluya. Esta discontinuidad en el flujo se produce, específicamente, en los profesionales de APS cuando, tras remitir al paciente a un nivel de mayor complejidad, las intervenciones y procesos en estos servicios difícilmente se conocen o, al retornar el paciente de la AE a la APS, el proceso e intervenciones hechas en la AE son conocidas por los profesionales de APS a través de la información del propio paciente y no por herramientas para la continuación y comunicación interna entre profesionales interniveles, lo que dificulta la continuidad en el proceso de atención:

*“[...] remitimos demasiados usuarios a un nivel superior pero no se nos retribuye la información, entonces hay cosas que uno se queda con mucha incógnita, precisamente es porque no hay esa cultura de comunicación entre niveles”: E2.*

Respecto a los limitantes administrativos, comprendiendo a priori el sistema de salud colombiano como una estructura tercerizada, desencadenan que el tiempo de espera de la asignación de citas, cumplimiento de las mismas y todas las atenciones necesarias en salud se prolonguen, implicando la dilatación del proceso sanitario, menos intervención en cantidad y calidad a los pacientes según su contexto, dificultando el seguimiento adecuado en ellos:

*“[…] Definitivamente no se está haciendo un seguimiento a los pacientes: llamar al paciente, identificar signos de alarma sobre la patología o sobre los procedimientos que se le hacen; eso no se ve ni en el medio hospitalario, ni se percibe a nivel de las Entidades Promotoras de Salud (EPS)”: E5.*

### Percepción de los enfermeros con relación a la satisfacción de necesidades del paciente con la Continuidad de Cuidados

Como ya se mencionó en la categoría anterior, en Colombia la aplicación de la CC es fragmentada, lo que produce que la percepción de los profesionales de enfermería respecto a la satisfacción de los pacientes con la aplicación de la CC.

Por lo tanto, la satisfacción de las necesidades de cuidado del paciente, desde la perspectiva de los profesionales de enfermería, se evidencia en el reconocimiento de estos en ambientes extrahospitalarios, en manifestaciones de agradecimiento a los profesionales:

*“[...] estaba en un evento y muchas mamás me estaban saludando, entonces una señora que estaba conmigo me decía ¿venga usted qué es que yo veo que la saludan tanto? Y yo: Es que yo soy enfermera y todas esas señoras son mamás de niños que yo tuve hospitalizados”: E1.*

Este fenómeno, según lo manifestado por los participantes, se debe a la relación humanística entre enfermero-paciente que facilita la confianza y calidez:

*“[…] en nosotros ven que hay un nivel de confianza más grande, lo que hace que el paciente se sincere mejor respecto a otros profesionales”. E2.*

Este fenómeno se manifiesta tanto en APS como en AE:

*“[…] Las mamás vienen y lo buscan a uno, entonces yo les dejo mi número y datos para la resolución de dudas […] para uno es muy satisfactorio, los pacientes ven que uno se está preocupando mucho más allá de una estancia hospitalaria”: E1. La preferencia por el profesional de enfermería la remiten, también, a la educación brindada dado su perfil profesional:“[…] quien mejor educa, hoy y siempre, ha sido el personal de enfermería, entrenado para hacer esa labor”: E1.*

La percepción de los enfermeros sobre la satisfacción del usuario con la práctica enfermera enmarcada en la CC, se manifiesta cuando los participantes verbalizan que el paciente puede sentirse satisfecho con la atención brindada por enfermería:

*“[…] Los pacientes sienten que se suplieron las necesidades porque ven las actividades de prevención desarrolladas por nosotros, además a ellos se sienten satisfechos cuando son escuchados, cuando los miren a los ojos, así sienten que enfermería si los está acompañando en su proceso de salud - enfermedad”: E6.*

Adicionalmente, la administración de la salud se percibe como una barrera en el cumplimiento satisfactorio para los usuarios por la dilatación de los procesos:

*“Hay necesidades que se suplen, pero hay otras que no, por las mismas complicaciones con los trámites administrativos, por las dificultades con las EPS, porque en muchas ocasiones tenemos pacientes que están a la espera de un procedimiento y la EPS no nos autoriza. Entonces llevamos quince días con el paciente a la espera, procurando manejar su patología, que salga por lo menos estable para darle manejo ambulatorio porque definitivamente no nos da para darle más manejo hospitalario”. E5.*

Relación de la percepción individual y la percepción colectiva sobre la Continuidad de Cuidados.

En las expresiones de los profesionales de enfermería se evidencia que la percepción individual de la práctica enfermera fundamentada en la CC no es practicada de forma organizada ni establecida por protocolos o guías.

La percepción colectiva apunta a que el seguimiento se realiza a los pacientes cuando existe un factor de riesgo, como por ejemplo las gestantes:

*“[…] Los médicos cuentan con un formato en el consultorio, yo lo diseñe para que esas pacientes que son de alto riesgo me las notifiquen, y yo con eso puedo hacerle seguimiento con la auxiliar”: E3. Asimismo, se manifiesta el seguimiento más riguroso a los infantes si presentan un factor de riesgo: “Si es un niño con desnutrición, se notifica al área de epidemiología y le hacen el seguimiento entes externos que tienen enfermeras encargadas de hacerle el seguimiento directamente”: E3.*

Se resalta que los participantes consideran importante vincular a la familia en el cuidado de procesos crónicos, por el riesgo de complicaciones y la necesidad de hacer cambios en los estilos de vida. Pero en el caso de los procesos agudos, la integración de la familia es mínima, debido a la rapidez del tratamiento, recuperación y egreso del mismo:

*“[…] no se vincule la familia es porque no son enfermedades que me van a generar un daño a largo plazo, yo solamente atiendo la enfermedad inicial, en el momento que atiendo el paciente, pero no estamos educando las familias para que esto después no se pueda presentar o las repercusiones que traería”: E4.*

En cuanto al entorno, los profesionales de enfermería afirman que los servicios de salud no solo se brindan en una institución reconocida, sino en diferentes contextos como colegios, domicilios, centros comerciales y parques:

*“[…] en nuestra profesión nosotros no la ejercemos solamente dentro de las instituciones de salud, la profesión se ejerce en diferentes ámbitos […] hay otros centros, colegios hasta en los centros comerciales, siempre hay un punto de enfermería donde se puede brindar una información que viene siendo educación.”: E4. “Estamos en la obligación de ir al domicilio porque tenemos que hacerle seguimiento a todos los casos especiales, estamos en la obligación de ir donde están las poblaciones cautivas: colegios, hogares, ancianatos, hogares de paso, todo lo que tenga que ver con este tipo de población porque ellos no pueden salir por unas condiciones especiales”: E2.*

Por otro lado, la información consideran importante darla al paciente y familia, sin embargo, esta se da a través de una gama de estrategias educativas desde un enfoque únicamente verbal:

*“[…] Claro, hay que buscar estrategias educativas que brinden una educación adecuada o sea que yo realmente me haga entender que los conceptos que yo tenga sobre la enfermedad sean totalmente entendidos por el paciente y su familia, para que la enfermedad no se vuelva a presentar en él ni a los que a él lo rodean” : E4*

Por último, la colectividad expresa que las herramientas de CC utilizadas en su contexto son principalmente los informes e historia clínica por procesos de acreditación institucional:

*“[…] acreditación es uno de los que exige como tal el plan de egreso del paciente, de pronto por estos momentos se esté hablando más acerca del plan de egreso y la vigilancia y el seguimiento del paciente dentro de habilitación”: E5. De igual forma, se hace uso de la historia clínica de forma estandarizada en las instituciones de salud o, en otros casos adicionales, formatos de seguimiento por clasificación de riesgo de los usuarios.*

## Discusión

El desconocimiento de la CC en el contexto colombiano es una realidad, no obstante, se aplican algunos pilares fundamentales como la información y contemplación de entornos diferentes al hospitalario como medios de prestación de los servicios de salud. Se evidencia la necesidad de modelos de atención dirigidos a la comunicación, trabajo en equipo y apoyo al autocuidado como mecanismo de atención integral que disminuya las atenciones de salud evitables.

Como respuesta a esto, las investigaciones españolas señalan la efectividad de distintas herramientas para la integración de la CC como la hospitalización domiciliaria y los coordinadores del cuidado que redujeron los reingresos de pacientes, la mortalidad y los días de hospitalización, mejoras en la calidad de vida y la satisfacción con la información recibida^2^^,^^15^^,^^16^^,^^21^^,^^22^^,^^23^. Esto evidencia las necesidades de los usuarios de un modelo de atención diferente a los utilizados hasta ahora ya que reportan falencias y dificultades en la misma. Se plantea la CC como un modelo de atención con mejores resultados, la cual puede aportar grandes beneficios en la atención de los colombianos en tiempos de pandemia y pos-pandemia.

La deficiente comunicación interniveles e interdisciplinaria reporta disminución en la percepción de la calidad de la atención brindada por el sistema de salud. En esta misma línea de ideas, enfermeras españolas manifiestan el poco registro de sus colegas en la historia clínica lo que dificulta el seguimiento del proceso del paciente^24^. Empero, la comunicación asertiva evidencia mejoras en la atención por la comunicación interinstitucional que permite el aumento en la CC como se evidencia en la atención al binomio madre-hijo con diagnóstico materno de VIH y disminución de la infección por este virus en los infantes^25^.

También, el reconocimiento del uso del plan de alta como cuidado transicional permite asegurar la coordinación y CC entre distintos niveles^3^. Darle la importancia a la educación a pacientes, familiares y cuidadores además de la transferencia de los cuidados dados, independientemente del nivel, aporta una postura fundamental a la hora de conseguir el mejor enfoque en la atención del paciente y la contextualización de los cuidados ofrecidos para la adherencia a este y la mejora en la autonomía y satisfacción de los pacientes respecto a su proceso de salud -enfermedad.

Si bien el modelo de atención en salud colombiano está trascendiendo con la Política de Atención Integral en Salud (PAIS), la cual tiene como objetivo la integralidad que comprende la igualdad de trato y oportunidades en el acceso y el abordaje integral de la salud y la enfermedad, al consolidar las actividades de promoción, prevención, diagnóstico, tratamiento, rehabilitación y paliación para todas las personas^11^; la percepción de la práctica y la realidad del sistema de salud colombiano se reconoce como causal de la dilación del acceso al sistema y la calidad percibida fluctúa. En este sentido, el cambio de paradigma en la prestación de servicios es una necesidad apremiante.

Puede surgir como una alternativa la tele enfermería, en el seguimiento del cuidado a las personas y/o comunidades, como motor agilizador del sistema, mecanismo de fortalecimiento de la atención y generador de oportunidades para el avance de enfermería, como pasa en algunos países como Brasil, costa rica, chile^26^^,^^27^. Con esto y la transformación de valores corporativos como la mutación a culturas organizacionales orientadas al grupo tienen mejores resultados en la implementación de la CC respecto a otro tipo de culturas organizacionales^28^. Se sugiere, por lo tanto, la imperiosa necesidad de la generación de estrategias políticas y sociales, con reestructuración del modelo de atención en salud que permita el avance de la disciplina enfermera en Colombia a un cuidado continuado y de calidad como lo propende la CC.

Al obtenerse la verbalización de la satisfacción de los sujetos de cuidado percibida por los enfermeros como un fenómeno divergente, la experiencia ibérica muestra que a pesar de ser poca la evidencia conseguida con relación a esta temática, la existente tiene la suficiente validez metodológica para ser un buen indicador de los beneficios que conlleva la aplicación de esta CC en los pacientes, no solo en su satisfacción personal, sino también en su calidad de vida; traducido esto en un mayor reconocimiento al profesional de enfermería y el rol que desempeña. Asimismo, la disminución de las re- consultas a los servicios de urgencias por exacerbación de la patología y la identificación de los signos y síntomas de la misma^22^^,^^7^.

La percepción individual con relación a la percepción colectiva determinó que los profesionales de enfermería estudiados no practican de manera organizada y completa la CC, evidenciado en el seguimiento en caso de existir un riesgo en salud. Además, afirman involucrar a la familia cuando se trata de pacientes con patologías crónicas, por su alta probabilidad de complicaciones, sin embargo, si es un proceso agudo consideran que no es necesario por su rapidez en la evolución y egreso del paciente.

En contraposición, la evidencia española refleja que la mayoría de los profesionales de enfermería realizan la continuidad asistencial de la atención primaria en salud al alta, basándose en protocolos y guías de entrevista, que tienen por objeto hacer una evaluación clínica y social para verificar la red de apoyo familiar, la capacidad de autocuidado, gestión terapéutica y grado de dependencia, esto permite que haya mayor calidad, seguridad y satisfacción de la atención^7^^,^^23^.

Con relación a la información, los profesionales de enfermería manifiestan que su realización se hace de forma verbal exclusivamente. Este punto fue contemplado en un estudio realizado en Portugal, resaltando que la continuidad de la atención abarca un conjunto de dimensiones que favorece la mejora en la atención sanitaria y posterior satisfacción de la atención, entre ellos está la información que los profesionales le trasmiten al sujeto de cuidado, la cual debe ser clara y suficiente^8^; sin embargo, aunque no se especifique la necesidad de emplear tanto la información verbal como escrita, se incluye como un pilar fundamental para la satisfacción de los pacientes con la CC.

El entorno en el que se genera la CC es muy diverso, debido a que los participantes reconocen que la atención en salud no solo se presta en una institución reconocida sino en diferentes contextos donde sea necesario, es por ello que los profesionales deben estar preparados para esto, como lo expresa la investigación costarricense, la cual demuestra que los profesionales de enfermería son capaces de romper barreras geográficas y de aprovechar los avances de la tecnología para mejorar el acceso a las cuidados y mejorar la calidad y continuidad de los mismos independientemente de la distancia, teniendo presente la capacitación, conocimiento y equipos suficientes ^3^^,^^26^^,^^27^.

Es de resaltar que la implementación de los componentes de la CC nombrados con anterioridad fue hecha con mayor énfasis por los coordinadores de los servicios comunitarios y el administrativo. En la realidad clínica, el cumplimiento de los ítems nombrados se dificulta, según lo verbalizado por los entrevistados, por la rapidez en la atención, aunque como los participantes lo manifestaron, se implementan algunos de los pasos de la CC con miras a la acreditación de alta calidad en los servicios.

Por último, con relación a las herramientas de seguimiento, los participantes solo utilizan los informes, la historia clínica o formatos de seguimiento por riesgo. Referido a este punto, la creación de la herramienta CTM-15 (Care Transitions Measure, por sus siglas en inglés), diseñada explícitamente para abarcar la experiencia global de la transición de cuidados en quince cuestiones, pasa a ser CTM-3, para poder incluir, además de algunos ítems relacionados con la CC, abordar la calidad de esta continuidad desde la perspectiva del paciente y el cuidador. Con esto, el cuestionario dejó de tener una orientación exclusivamente médica y permitió medir la satisfacción del paciente con la continuidad interniveles que ofrecen los profesionales de enfermería y estableció que los pacientes con informe de cuidados tienen un nivel más alto de satisfacción con la CC^29^.

Por consiguiente, es necesario contar con seguimiento fundamentado, con el fin de obtener resultados positivos en los pacientes, para contribuir así al autocuidado de los mismos y autonomía de los profesionales de enfermería y los sujetos de cuidado.

Contribución de la investigación: La contribución de la investigación le apunta al aterrizaje de la necesidad de la implementación de la CC en la práctica enfermera colombiana. La evidencia científica sobre los beneficios del uso de la CC tanto para la administración sanitaria, profesionales y sujetos de cuidado son amplias y cada vez con mayor afluencia desde el conocimiento del proceso de la CC. Los diferentes componentes que conforman la CC no son ajenos al contexto colombiano: personas, entorno e información; por lo que la reestructuración y organización de estos traería grandes beneficios en la efectividad de la red de salud colombiana.

Adicionalmente, para el tiempo pandémico que se llegó con la diseminación de la infección por SARS-COV 2, la tele-salud floreciente por las medidas de bioseguridad se puede contemplar desde la CC como proveedora de seguimiento domiciliario a la comunidad en aras de un control de la evolución de su salud y disminución de la circulación viral comunitaria. Asimismo, la tele-salud puede permitir la mejora de la respuesta de la atención primaria a la pandemia y descongestión del nivel especializado del sistema salud, situación a explorar en tiempos de pandemia, que podría extenderse a un seguimiento de pacientes con cualquier tipo de necesidad de continuidad.

## Conclusiones

En conclusión, la necesidad de la información brindada al paciente, cuidador, familiar y/o población resalta la importancia de ofrecer más que información, educación verídica, científica y útil, ayudando a descartar fuentes de información falsa o poco confiable que creen angustia, ansiedad y dificultad en la adherencia al plan de atención. Esto favorece la autonomía en la toma de decisiones en materia de salud sobre todo de pacientes y cuidadores.

Los resultados indican que la práctica de la CC es fragmentado y empírica, obteniendo así alguna mejora en la condición de salud del paciente; si la CC se practicara integralmente en el ciclo de vida de la persona y transversalmente en el Sistema de Salud, se mejoraría la calidad tanto del servicio prestado como de vida de la población intervenida, además de proveer de un marco de respuesta para el período pos-pandemia.

Según lo manifestado por los participantes, la CC no se transmite como conocimiento epistemológico en los planes de estudios de los pregrados en Colombia, situación que se transforma en un motivo de dificultad en su aplicabilidad en el territorio.

Además, se enfatiza que, para lograr la satisfacción y mejora de la calidad de vida de los sujetos de cuidado a través de la aplicación de la CC, se requiere una acción interdisciplinaria. Lo que pone en evidencia la necesidad de una integración entre profesionales de medicina, trabajo social, gerontología, psicología, odontología, nutrición, entre otros; y enfermería para dar respuesta al cumplimiento de la CC, siendo enfáticos en que la responsabilidad y éxito de la CC recae sobre este último profesional.

Limitaciones: El acceso de los investigadores a los participantes e instituciones fue laborioso, además de la falta de antecedentes investigativos en Colombia sobre el tema abordado. Se contempló como una oportunidad para identificar nuevas lagunas en la literatura y, consecuentemente, surgió esta investigación.

Recomendaciones: Se recomienda ampliar la valoración de la aplicabilidad de la CC en diferentes contextos del territorio colombiano como método de establecimiento de un panorama general del fenómeno, creando antecedentes que validen la reorganización de la práctica enfermera en el contexto nacional.

De igual forma, se sugiere evaluar la satisfacción de los sujetos de cuidado respecto a la práctica enfermera desde la perspectiva de ellos, como medio de contraste de los resultados obtenidos en esta investigación de la satisfacción del usuario desde la perspectiva del profesional de enfermería.

Por último, la aplicación de los instrumentos de CC para la posterior medición del grado de continuidad, con miras a evaluar el cumplimiento y expectativas respecto a esta, pudiendo evaluarse en diferentes contextos y con enfoques cualitativos.

## References

[1] Sánchez BG., Martínez Riera JR, del Pino Casado R (2014). Manual práctico de enfermería comunitaria.

[2] Ramírez-Girón N, Cortés-Barragán B, Galicia-Aguilar RM (2016). Continuidad del cuidado: adulto mayor con diabetes tipo 2 y su cuidador. Enferm. univ..

[3] Utzumi FC, Lacerda MR, Elizabeth B, Gomes IM, Aued GK, Sousa SM (2018). Continuidade do cuidado e o interacionismo simbólico: um entendimento possível. Texto & Contexto - Enfermagem.

[4] Berenguer-García N, Roldán-Chicano MT, Rodríguez-Tello J, García-López MM, Dávila- Martínez R, Bueno-García MJ (2018). Validación del cuestionario CTM-3-modificado sobre satisfacción con la continuidad de cuidados: un estudio de cohortes. Aquichan.

[5] Fátima Soza Diaz Clarivel De, Luz Bazán Sánchez Asunción Carmen, Jeuna Diaz Manchay Rosa (2020). Percepción de las enfermeras sobre el uso de sus registros para garantizar la continuidad del cuidado.

[6] Guzmán Magaly Del Carmen Gallardo, Ferreira Alexandra, Andrade Selma Regina de (2020). Role of nurses for continuity of care after hospital discharge. Texto & Contexto - Enfermagem.

[7] Costa MFBNA da, Andrade SR de, Soares CF, Pérez EIB, Tomás SC, Bernardino E (2019). The continuity of hospital nursing care for Primary Health Care in Spain. Revista da Escola de Enfermagem da U S P.

[8] Mendes FRP, Gemito MLGP, Caldeira E do C, Serra I da C, Casas-Novas MV (2017). A continuidade de cuidados de saúde na perspetiva dos utentes. Ciênc. Saude colet..

[9] Puello Ortega A, Prieto Silva R, Rondón Medina V (2016). Comunicación y coordinación en enfermería en una institución de salud de alta complejidad en Cartagena. Av en Enferm..

[10] Martí Montserrat Pérez (2017). Percepción de los/as enfermeros/as sobre el Informe de Cuidados de Enfermería al Alta en un Hospital Comarcal. NURE Investigación.

[11] Ministerio de Salud y Protección Social (2016). Política de Atención Integral en Salud Ministerio de Salud y Protección Social.

[12] ConsejoTécnico Nacional de Enfermería (2019). Plan nacional de Enfermería 2020-2030 Fortalecimiento de la profesión de Enfermería en Colombia.

[13] Congreso de Colombia (1996). Ley 266 de 1996. Diario Oficial No. 42.710.

[14] Congreso de Colombia (2004). Ley 911 de 2004. Diario Oficial No. 45.693.

[15] Rodríguez AG (2011). Programas de continuidad de cuidados: éxitos, fracasos y retos futuros. Estud Psicol..

[16] Vargas Melgarejo LM. (1994). Alteridades.

[17] Alonso Dos Santos M. (2016). Calidad y satisfacción: el caso de la Universidad de Jaén. Rev. educ. sup..

[18] Polit D, Beck CT. (2018). Fundamentos da pesquisa em enfermagem: avaliação de evidências para a prática de enfermagem.

[19] Bardin L., Rego L. de A., Pinheiro A. (2016). Análise de conteúdo.

[20] Minayo MC. (2014). O Desafio do Conhecimento: Pesquisa qualitativa em saúde.

[21] Fernández AM, Machín JM, Martín MD, Aller MB, Vallejo IM (2017). Care models for polypathological patients. Rev Clin Esp (Barc).

[22] Li SX, Del Carmen MG, Thompson RW, Cafiero-Fonseca ET, Rockett H, Ferris TG (2021). Evaluation of Hospitalizations Preventable with Idealized Outpatient Care and Continuity of Care. J Healthc Qual..

[23] Corral LG, Cengotita-Bengoa MB, Sánchez RJ, García JA (2017). El paciente y su travesía entre la atención primaria y la hospitalaria. Revisión sistemática de ensayos clínicos para la implementación de herramientas para la integración en España. Anales Sis San Navarra.

[24] Rios AM, Artigas ML, Sancho MG, Blanco CA, Acedo MA, Calvet GT (2020). Standardized nursing languages and care plans. Perception of use and utility in primary healthcare. Atencion primaria.

[25] McNairy ML, Teasdale CA, El-Sadr WM, Mave V, Abrams EJ (2015). Mother and child both matter: reconceptualizing the prevention of mother-to-child transmission care continuum. Curr Opin HIV AIDS..

[26] Hung D, Chung S, Martinez M, Tai-Seale M (2016). Effect of Organizational Culture on Patient Access, Care Continuity, and Experience of Primary Care. J Ambul Care Manage.

[27] Toffoletto MC, Ahumada-Tello JD (2020). Telenursing in care, education and management in Latin America and the Caribbean: an integrative review. Rev Bras Enferm..

[28] Carvajal Flores L, Vásquez Vargas L (2016). Conocimiento, PrácticaY Percepción De Enfermeras Respecto A Tele-Enfermería Como Estrategia De Continuidad Del Cuidado. Enfermería.

[29] Berenguer-García N, Roldán-Chicano MT, Rodriguez-Tello J, García-López M del M, Dávila-Martínez R, Bueno-García MJ (2018). Validación del cuestionario CTM-3-modifi cado sobre satisfacción con la continuidad de cuidados: un estudio de cohortes. Aquichan.

